# The Driving Profile of Individuals with Schizophrenia: Cognitive Characteristics, Pharmacological Treatment and Driving Competence—A Scoping Review

**DOI:** 10.3390/neurolint18030046

**Published:** 2026-02-28

**Authors:** Elpida Stratou, Georgia-Nektaria Porfyri, Aikaterini Gamvroula, Katerina Theodorou, Symeon Dimitrios Daskalou, Nikolaos Gerosideris, Georgia Tsakni, Foteini Christidi, Anna Tsiakiri, Pinelopi Vlotinou, Ioanna Giannoula Katsouri

**Affiliations:** 1 Department of Occupational Therapy, University of West Attica, 12243 Egaleo, Greece; sdaskalou@uniwa.gr (S.D.D.); ngerosideris@uniwa.gr (N.G.); ytsakni@uniwa.gr (G.T.); pvlotinou@uniwa.gr (P.V.); ykatsouri@uniwa.gr (I.G.K.); 2 Department of Psychiatry, General Hospital of Argolida, 21231 Argos, Greece; geoporfyri@hotmail.fr (G.-N.P.); gamvroulak@gmail.com (A.G.); katerina_mateika@hotmail.com (K.T.); 3 Department of Psychology, School of Philosophy, National and Kapodistrian University of Athens, 15784 Athens, Greece; fchristidi@med.uoa.gr; 4 Neurology Department, Democritus University of Thrace, 68100 Alexandroupolis, Greece; atsiakir@med.duth.gr

**Keywords:** schizophrenia, driving, driving profile, medication adherence, cognitive performance, functional capacity

## Abstract

**Background/Objectives**: Driving performance and competence represent a complex functional domain that may be affected in some individuals with schizophrenia. This scoping review aimed to map existing evidence characterizing driving-related functioning by identifying the cognitive, pharmacological and functional factors that influence driving ability and by synthesizing findings from experimental, neurocognitive and population-based studies. **Methods**: A structured search of the PubMed, Scopus and ScienceDirect databases was performed in accordance with PRISMA-ScR guidelines to identify studies published between 2015 and 2025 that examined cognitive, pharmacological and functional dimensions of driving in individuals with schizophrenia. Extracted data were narratively and thematically synthesized. Eleven studies met the inclusion criteria. **Results**: Findings clustered into three domains: cognitive, including attention, executive function, reaction time and visuospatial processing; pharmacological, encompassing drug comparisons, dosage, side effects and treatment stability; and functional, covering license status, driving participation, driving cessation, avoidance behaviors and self-regulation. **Conclusions**: This review integrates current evidence within a multidimensional and conditional framework, highlighting interactions between cognitive functioning, pharmacological factors, and compensatory self-regulation in individuals with schizophrenia. Understanding these interrelations may inform individualized fitness-to-drive evaluations and contribute to structured, context-sensitive interpretation of driving-related evidence in clinical and regulatory settings.

## 1. Introduction

Schizophrenia affects approximately 23–24 million people worldwide and is associated with substantial disability and functional impairment [1]. It is characterized by positive symptoms such as hallucinations and delusions, as well as negative and cognitive deficits, including impaired executive function, attention, and processing speed [2]. In addition to schizophrenia’s psychotic and cognitive features, its pharmacological treatment and associated side effects, such as sedation and extrapyramidal symptoms, can further influence individuals’ ability to perform complex everyday tasks, including driving [3,4,5,6,7].

Epidemiological evidence suggests that individuals with schizophrenia are less likely to hold a driver’s license and may exhibit higher rates of driving cessation or crash involvement compared to the general population [4,5,6,8,9]. In some jurisdictions, a diagnosis of schizophrenia may trigger restrictions or mandatory fitness-to-drive evaluations [3]. However, systematic reviews indicate that, under conditions of clinical stability and treatment adherence, many individuals with schizophrenia can drive safely [10,11,12,13]. Moreover, several experimental and simulator-based studies have reported comparable braking responses, lane control, and collision rates between individuals with schizophrenia and healthy controls [4,7,14,15,16]. Beyond diagnostic status, research in the general population highlights the influence of self-perceived ability and risk-taking behavior on driving performance [17].

Conflicting data highlight the complexity of this topic and the fact that, despite numerous existing studies, no synthesis has systematically structured the multidimensional determinants shaping driving-related functioning in schizophrenia across cognitive, behavioral, neurophysiological and population-level evidence. Systematic reviews have addressed individual domains, including neurocognitive correlates [13], medication-related driving performance [12], and crash risk in psychiatric populations [11]. Experimental studies have also examined associations among cognitive function, symptom severity, and simulated driving performance, as shown by Okada et al. (2024) [18]. However, these domains remain fragmented, and an integrative framework that situates clinical, experimental, and epidemiological findings within a structured conceptual framework is still lacking.

Rather than assuming a homogeneous phenotype, existing evidence points to substantial inter-individual variability shaped by clinical stability, cognitive functioning, treatment characteristics, and contextual factors [7]. A structured conceptual framework may therefore help organize these interacting determinants while explicitly accommodating heterogeneity and avoiding deterministic classifications of individuals as categorically fit or unfit to drive.

We should keep in mind that driving is not merely a transportation skill but a crucial element of autonomy, self-esteem, and community participation [3,7,15]. It represents an important aspect of independence and social integration, particularly in the context of mental health conditions [9,19]. However, although driving is a key element in patients’ recovery-oriented functioning and social reintegration, it remains a hard topic for discussion, often surrounded by stigma, even for healthcare workers [19]. On the contrary, patients want to explore every aspect of this topic, asking for support and direct conversations about medication side effects and their impact on driving ability, as well as strategies to gain their license and maintain or return to driving [20]. Given the ethical implications of misclassification and diagnostic overgeneralization, a non-deterministic and individualized approach to interpreting driving-related evidence is warranted.

In the present review, driving ability is conceptualized as a multidimensional and conditional construct, encompassing cognitive and psychomotor capacity, structured driving performance, real-world participation, and safety-related outcomes. These domains are treated not as interchangeable indicators of driving ability, but as analytically distinct levels within a structured hierarchy, each reflecting different aspects of functioning and requiring separate interpretation. This distinction is consistent with the International Classification of Functioning (ICF) [21], which differentiates body functions, activity performance, and participation. Within the ICF framework, impairments at the level of body functions do not automatically translate into activity limitations or participation restrictions, as contextual and environmental factors may moderate functional expression. This conceptualization is consistent with established hierarchical and self-regulation models of driving behavior, including Michon’s hierarchical model [22] and the Task–Capability Interface Model [23], which distinguish between operational performance, tactical regulation, and broader participation-level processes.

In this direction, the present scoping review aims to delineate and map the cognitive, pharmacological, and functional domains associated with driving-related functioning in individuals with schizophrenia by synthesizing findings from experimental, neurocognitive and population-based studies. The objective is not to define a single unified driving phenotype, but to provide a heterogeneity-compatible conceptual framework that may provide a structured basis for future hypothesis-driven research and for cautious, context-sensitive interpretation of driving-related evidence within clinical and regulatory discussions, without implying prescriptive recommendations.

## 2. Materials and Methods

### 2.1. Transparency and Openness

This scoping review was conducted in line with established methodological frameworks [24,25] and the Joanna Briggs Institute guidance [26]. Reporting followed the PRISMA Extension for Scoping Reviews (PRISMA-ScR) to ensure transparency and consistency [27]. This scoping review was retrospectively registered on the Open Science Framework (OSF; https://doi.org/10.17605/OSF.IO/FCZEG). The complete PRISMA checklist is available as Supplementary Material (Supplementary File S1)

### 2.2. Scoping Review Research Questions

The scoping review focused on addressing three key research questions:

(1) Which cognitive, pharmacological, and functional domains are associated with driving-related functioning in individuals with schizophrenia?
(2) How are these domains conceptually and empirically related to driving-related outcomes, including simulated and real-world measures?
(3) What methodological or conceptual gaps remain in defining this profile across the existing literature?

### 2.3. Search Strategy

Relevant studies were identified through systematic searches of the PubMed, Scopus, and ScienceDirect databases. The searches covered the period from January 2015 to September 2025. This timeframe was selected to capture contemporary evidence reflecting advances in high-fidelity driving simulator methodologies, the integration of neuroimaging techniques in driving research, and recent population-based crash registry analyses, as well as current antipsychotic treatment practices. Focusing on the past decade allowed the review to synthesize findings aligned with contemporary clinical, technological, and regulatory contexts. The search strategy combined medical subject headings and free-text terms related to schizophrenia, driving, and medication adherence. Keywords and search terms included combinations of “schizophrenia,” “psychosis,” “driving competence,” “fitness to drive,” “cognitive function,” “executive function,” “attention,” “visual perception,” “psychomotor performance,” “simulator,” “medication adherence,” “side effects” and “antipsychotic treatment”. Search strings were adapted to the indexing systems of each database.

### 2.4. Eligibility Criteria

The eligibility criteria for this review were defined in accordance with the Population–Concept–Context (PCC) approach as outlined in the Joanna Briggs Institute guidance [26]. The population of interest comprised adults diagnosed with schizophrenia, regardless of illness duration or treatment setting. The concept focused on driving ability or driving-related competencies, including both real-world indicators and experimental measures from driving simulators, neuropsychological tests, self-reported driving behavior, and neuroimaging tasks. The context encompassed research examining clinical, cognitive, pharmacological, or functional factors influencing driving, including the effects of antipsychotic medication, side effects, and adherence. Only peer-reviewed studies published in English were included, consistent with feasibility constraints and common practice in scoping reviews. All study designs were considered eligible, provided they offered relevant data for mapping the driving profile in schizophrenia.

### 2.5. Study Selection and Data Extraction

**Figure 1:**
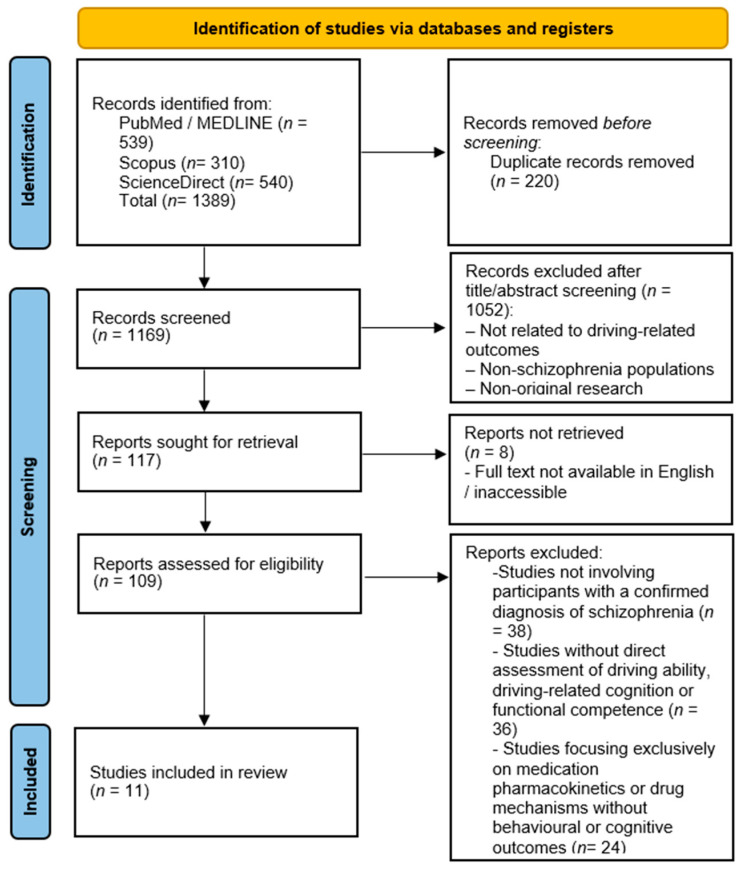
Flowchart of the literature search and study selection process according to the PRISMA-ScR guidelines.

The study selection process followed the PRISMA-ScR flow diagram [27] (Figure 1), illustrating the identification, screening, eligibility, and inclusion of studies. Titles and abstracts were initially screened for relevance, followed by a full-text review of potentially eligible articles. Any disagreements were resolved through discussion among the reviewers to ensure methodological consistency and transparency. Data extracted from each study included study design and methodological characteristics, sample demographics and clinical profile, driving-related outcomes and assessment methods, as well as cognitive, pharmacological and functional indicators contributing to the overall driving profile. The extracted data were organized in structured tables to enable comparison across studies. Finally, the data were collated, summarized, and synthesized thematically, with particular attention to (a) clinical and behavioral factors examined in relation to driving-related functioning, (b) cognitive and neuropsychological correlates of driving performance, (c) pharmacological and treatment-related characteristics reported in association with driving outcomes, and (d) functional capacity, self-regulation, and real-world driving measures. Consistent with the objectives of scoping review methodology, the review employed narrative and thematic synthesis rather than quantitative pooling. The heterogeneity of study designs, outcome definitions, and effect measures further supported this approach.

## 3. Results

### 3.1. Study Characteristics

A total of 11 studies published between 2015 and 2025 were included in this scoping review. Study designs varied substantially, encompassing cross-sectional surveys and interviews [6,7,8,9,28], comparative and experimental studies employing neuropsychological, cognitive and functional testing, driving simulators, or neuroimaging methods [14,16,18,29], as well as population-based cohort and case-crossover analyses using linked health and traffic records [4,5]. Sample sizes varied considerably across studies, ranging from small laboratory-based samples of 20–60 participants [14,16,18,29] to larger clinical and outpatient cohorts of approximately 50–150 individuals [6,7,8,9,28]. The largest datasets were represented by population-level analyses, including over 800,000 licensed drivers in British Columbia [5] and more than 1100 crash-involved drivers with schizophrenia [4]. Across the smaller-scale studies, samples often included either people with schizophrenia and healthy controls [14,16] or subgroups defined by treatment type, illness stage, or license status [25,29]. Clinical populations were typically composed of outpatients or clinically stable individuals [6,7], while some studies included psychiatric inpatients or broader psychiatric cohorts for comparison [8]. This methodological diversity provided complementary perspectives on cognitive, pharmacological, and functional dimensions relevant to driving-related functioning. A summary of study characteristics and key findings is presented in Table 1.

To enhance conceptual clarity, the findings were analytically interpreted across distinct but interrelated levels of driving ability: (a) cognitive and psychomotor capacity, (b) structured or simulator-based driving performance, (c) real-world driving participation (e.g., license possession and activity), and (d) safety-related outcomes such as crash involvement or responsibility. These levels correspond to the International Classification of Functioning, Disability and Health (ICF) [21], where body functions are related to activity performance and, subsequently, participation and real-world outcomes. The methodological grouping of studies described below aligns with these analytical levels but does not imply conceptual interchangeability among them.

From a methodological perspective, the included studies can be broadly classified into three complementary domains:

– Cognitive, psychomotor and functional performance investigations, employing simulator-based paradigms, reaction-time tasks, and comprehensive neuropsychological batteries to evaluate attention, perception, motor control, and executive functioning relevant to driving performance as domains examined in relation to driving competence [6,7,14,16,18,29].
– Clinical, pharmacological and functional correlates of driving participation were examined in studies focusing on mobility patterns, licensing status and the association of psychopathology, cognitive performance, and socio-demographic factors with real-world driving engagement [8,9,28].
– Population-based crash risk analyses utilizing large-scale administrative health and driving records to explore associations between schizophrenia, antipsychotic treatment adherence, and motor vehicle crash outcomes [4,5].

### 3.2. Driving-Related Measures and Assessment Approaches

Driving-related outcomes were assessed using a wide range of methodologies, reflecting methodological diversity across experimental and real-world contexts. Self-reported indicators of driver’s license possession, driving activity, accident history, and car use were used in survey-based and clinical studies [8,9,28]. These measures were often combined with standardized cognitive or functional assessments such as the Trail Making Test (TMT-A), Wisconsin Card Sorting Test (WCST), Mini-Mental State Examination (MMSE), and Global Assessment of Functioning (GAF) to examine factors reported in relation to driving participation and safety. Objective psychomotor and cognitive performance were examined through the Vienna Test System (WTS), following German traffic safety standards, including tests of visual perception (TAVT-MB), reactivity and stress tolerance (DT), concentration (COG), and vigilance (VIGIL), which were used to derive global impairment classifications within those studies [29]. Biedermann et al. [7] further integrated expert evaluations alongside WTS results to assess the combined associations of symptom severity, antipsychotic dosage, extrapyramidal side effects, and functional capacity with driving competence classifications.

Experimental and simulator-based studies [14,16,18] evaluated real-time driving behaviors, such as reaction to critical events, lane control, speed regulation, braking performance, and hazard perception. Fuermaier et al. [16] combined these outcomes with an extensive neuropsychological battery covering attention, working memory, inhibition, planning, and processing speed. Okada et al. [14] employed functional near-infrared spectroscopy (fNIRS) during simulated driving tasks to capture dorsolateral prefrontal cortex (DLPFC) activation, while Okada et al. [18] integrated neuropsychological tests (TMT-A/B, WMS-R, Zoo Map Test), fNIRS, and a detailed urban driving hazard prediction task, linking neural activation patterns to behavioral outcomes such as steering stability, braking efficiency and standard deviation of lateral position (SDLP). Population-level studies [4,5] operationalized driving outcomes through validated police crash data, focusing on crash responsibility and motor vehicle crash involvement among drivers with schizophrenia. Medication adherence was quantified using the Medication Possession Ratio (MPR), comparing medication coverage during pre-crash and control intervals.

### 3.3. Cognitive, Clinical, Pharmacological and Functional Correlates of Driving Performance in Schizophrenia

Cognitive, clinical, pharmacological, and functional factors were examined across studies in relation to driving-related functioning in individuals with schizophrenia across laboratory, simulator, and clinical settings. In a comparative psychomotor study, 58% of schizophrenia patients demonstrated impairments in driving-related skills, with first-episode participants showing additional deficits in visual perception compared to recurrent cases and healthy controls [29]. These deficits were reported as not significantly associated with medication status in that study and were not correlated with PANSS symptom severity. Similarly, simulator-based assessments revealed that schizophrenia patients did not exhibit higher collision rates but tended to drive more slowly and cautiously, often hindering merging vehicles. Cognitive measures of attention, inhibition, planning and processing speed were associated with lane control and reaction to critical events [16]. License-holding patients generally showed higher cognitive and executive performance. Individuals with a driver’s license had higher MMSE scores, better executive functioning on the WCST, and lower general psychopathology than non-drivers [28].

In real-world fitness assessments, higher antipsychotic dosage, older age, and extrapyramidal symptoms were reported to be associated with lower classifications of driving competence, whereas residual symptom dimensions, except disorganization, showed minimal associations [7]. The type of antipsychotic medication was also examined in relation to cognitive competence relevant for driving. In that sample, higher proportions of participants receiving aripiprazole or paliperidone were classified as competent to drive (approximately 75–82%) compared to those receiving haloperidol or risperidone (33–57%) [6].

Neurophysiological investigations added another layer of evidence. Functional near-infrared spectroscopy (fNIRS) during simulated driving tasks showed altered dorsolateral prefrontal cortex activation in schizophrenia participants under high-demand braking conditions, with reduced left DLPFC activity reported to be associated with slower reaction times. Combined neuropsychological testing and fNIRS measures were associated with hazard perception performance and classification of collision outcomes, with discriminant models achieving approximately 79% accuracy within the studied samples [14,18]. The cognitive, clinical, pharmacological, and functional domains reported across study designs are summarized in Table 2.

### 3.4. Driving Participation, Licensing and Mobility Outcomes

Rates of driver’s license possession and active driving were markedly lower among individuals with schizophrenia compared to control groups. In a clinical inpatient sample, only 67% held a driver’s license compared with 89% of neurological controls, with schizophrenia reported as being associated with lower levels of current driving activity. Female sex, older age, pension status, and schizophrenia diagnosis were identified as key predictors of driving cessation [8]. Consistent with these findings, another cross-sectional survey reported that although 64% of participants with schizophrenia or schizoaffective disorder possessed a valid license, only 32% had driven during the previous year. Cognitive functioning (Trail Making Test A), GAF scores, hospitalization frequency, and substance use history reported as significantly associated with active driving status [9]. Further support came from studies examining combined cognitive, clinical, and functional moderators of driving participation. License holders generally displayed higher cognitive performance, fewer hospitalizations, greater employment rates, and lower symptom severity, particularly on general and negative PANSS subscales, compared to non-license holders [28]. In medicated samples, patients’ fitness to drive varied according to antipsychotic type, with those receiving second-generation antipsychotics such as aripiprazole or paliperidone showing higher proportions of fitness-to-drive classifications within that sample compared to other treatments [6].

### 3.5. Crash Risk and Real-World Driving Safety

Large-scale epidemiological data have provided important insights into real-world crash risk among drivers with schizophrenia. In a population-based retrospective cohort study, Staples et al. [5] analyzed over 808,000 licensed drivers in British Columbia, including 2551 individuals with schizophrenia. Drivers with schizophrenia were significantly more likely to be deemed responsible for crashes compared with those without the disorder (66.2% vs. 53.7%; adjusted OR = 1.67), a magnitude comparable to other well-established predictors of crash risk, such as older age or learner license status. Notably, antipsychotic adherence defined as MPR ≥ 0.8 did not significantly alter the likelihood of crash responsibility, indicating no statistically significant association between adherence (as operationalized in that study) and crash responsibility. A subsequent case-crossover study by Staples et al. [4] examined 1130 police-attended crashes involving drivers with schizophrenia on outpatient antipsychotic treatment. Perfect adherence (MPR ≈ 1.0) was associated with a 50% reduction in crash odds compared with complete non-adherence (aOR = 0.50, 95% CI: 0.38–0.66), reflecting an observed association within the specified exposure window rather than establishing causality.

## 4. Discussion

### 4.1. Summary of Main Findings

This scoping review synthesized evidence from studies published between 2015 and 2025 that examined the clinical, cognitive, pharmacological, and functional determinants of driving fitness and safety in individuals with schizophrenia. Across methodologies ranging from cross-sectional surveys and neurocognitive assessments to experimental driving simulators and population-level crash data, findings generally suggest considerable inter-individual variability in driving performance among people with schizophrenia.

Specifically, cross-sectional surveys and structured interviews showed that license possession and active driving were lower among schizophrenia patients compared to controls, with predictors including age, sex, cognitive functioning, and clinical stability [8,9,28]. Importantly, license possession and driving participation reflect activity and participation-level indicators and should not be interpreted as direct measures of driving competence or safety. Comparative psychomotor assessments using standardized batteries revealed that first-episode and recurrent schizophrenia patients demonstrated significant impairments in visual perception, reaction time, concentration, and vigilance compared to healthy controls [29]. Experimental driving simulator studies have indicated that patients drive more slowly, have difficulty with merging, and exhibit impaired lane control, largely mediated by deficits in attention, inhibition, and planning [14,16,18]. These simulator-based findings represent structured performance-level outcomes and do not necessarily translate directly into real-world crash risk. Neurocognitive and functional assessments further highlighted that better executive function, higher MMSE scores, and fewer hospitalizations were associated with license possession and active driving [7,28].

Medication-specific effects were also observed, as summarized in Brunnauer et al. [12], with patients treated with second-generation antipsychotics such as aripiprazole and paliperidone were classified as competent at higher rates within the respective samples. However, these associations derive primarily from observational designs and may reflect underlying clinical characteristics, illness stage, or prescribing patterns rather than medication-specific causal effects. Functional capacity and clinical stability were consistently linked to safe driving; in a naturalistic cross-sectional study, 44% of clinically stable outpatients were deemed competent drivers, while age, extrapyramidal symptoms and antipsychotic dosage negatively affected driving fitness [7]. Such classifications, while clinically meaningful, represent context-dependent clinical judgments rather than standardized legal determinations of driving ability.

Driving simulator studies combined with neuroimaging revealed compensatory neural mechanisms, with reduced dorsolateral prefrontal activation correlating with slower reaction times during complex driving tasks [14,18]. These neurophysiological findings should be interpreted as correlational indicators within controlled experimental contexts, as their external predictive validity for real-world crash outcomes remains insufficiently established. Finally, population-based analyses indicated that although real-world crash responsibility was sometimes elevated among drivers with schizophrenia, adherence to antipsychotic medication, symptom stabilization, and compensatory self-regulatory behaviors may mitigate risk in some cases [4,5]. These findings indicate associations rather than causality and should be interpreted within the constraints of observational epidemiological designs. As a scoping review, the present study aimed to map and synthesize existing evidence rather than evaluate intervention effectiveness, establish causal relationships, or generate pooled effect estimates.

### 4.2. Comparison with Previous Literature

Our findings are largely consistent with prior systematic reviews, which highlight cognitive deficits as central determinants of driving competence in schizophrenia and other psychiatric populations [10,12]. Specifically, impairments in attention, executive functioning, processing speed, visual perception, and psychomotor skills observed in our included studies [6,7,8,9,14,16,28,29] mirror patterns reported in previous research, suggesting that residual cognitive dysfunction may persist despite pharmacological treatment in some individuals.

In other mental health conditions, including major depressive disorder and bipolar disorder, psychomotor slowing, fatigue and reduced self-insight similarly affect driving performance, reflecting the combined impact of illness-specific deficits, pharmacological effects, and compensatory strategies [10,11]. It should be noted that these are analogous observations and not direct evidence from schizophrenia populations. For instance, discontinuation of sedating medications or mood-stabilizing treatments has been linked to increased crash risk, while adherence to pharmacotherapy has been associated with differences in driving-related outcomes [11]. These comparisons are presented for contextual purposes and do not imply cross-diagnostic equivalence.

Neuroimaging and experimental studies provide additional insights into neural and behavioral mechanisms. Okada et al. [14,18] observed altered dorsolateral prefrontal activation and slower hazard perception in schizophrenia patients during driving simulations, suggesting compensatory strategies that may partially preserve performance. However, such findings derive from controlled experimental paradigms and their direct applicability to real-world driving safety remains to be established. Similarly, executive function and psychomotor speed have been associated with real-world driving performance in euthymic bipolar patients [11]. Population-level analyses further indicate statistical associations between functional capacity, treatment adherence, self-regulatory behaviors and crash-related outcomes [4,5,11], though causal pathways cannot be inferred from the available evidence.

### 4.3. Defining the Driving Profile of Individuals with Schizophrenia

Taken together, the evidence suggests that individuals with schizophrenia demonstrate patterns of driving-related functioning that can be described within a multidimensional and conditional framework, rather than exhibiting a single uniform driving phenotype. More specifically, first-episode and recurrent schizophrenia patients show significant impairments in visual perception, reaction time, concentration, and vigilance [26]. They drive more slowly, struggle with merging, and exhibit impaired lane control, largely mediated by deficits in attention, inhibition, and planning [16,18]. However, clinically stable outpatients may be deemed competent drivers, exhibit better executive function, higher MMSE scores, and have fewer hospitalizations [7].

Those treated with second-generation antipsychotics such as aripiprazole and paliperidone have been reported in some studies to demonstrate higher driving-related cognitive performance compared to individuals prescribed haloperidol or risperidone [6]; however, these findings may reflect differences in illness severity, functional baseline, or prescribing patterns rather than medication-specific causal effects. Adherence to antipsychotic medication, symptom stabilization and compensatory self-regulatory behaviors have been statistically associated with differences in crash-related outcomes in some population-based analyses, while age, extrapyramidal symptoms and antipsychotic dosage negatively affect driving fitness [5,7]. These associations should be interpreted within the constraints of observational and cross-sectional designs.

Accordingly, the proposed driving profile is conceptualized as a structured framework that organizes interacting cognitive, pharmacological, clinical, and functional determinants, while explicitly accommodating heterogeneity and contextual variability. This framework may assist clinicians and occupational assessors in organizing relevant domains for individualized evaluation, but it does not replace formal fitness-to-drive assessments nor imply deterministic classification of individuals as universally fit or unfit to drive.

### 4.4. Clinical and Functional Implications

These findings highlight the potential relevance of comprehensive and individualized considerations when evaluating driving-related functioning in schizophrenia and other psychiatric populations. Cognitive deficits, particularly in attention, executive function, processing speed, visual perception, and psychomotor performance, consistently correlate with simulator and on-road driving outcomes [7,11,14,16]. Within a structured assessment context, clinicians may consider integrating cognitive testing with evaluations of functional capacity, self-regulatory behaviors, insight, and psychosocial functioning when advising patients [3,8,11,28]. In addition to cognitive and functional considerations, pharmacological factors have been examined in relation to driving-related outcomes. Adherence to antipsychotic medication has been associated in some studies with differences in crash-related outcomes, though it does not fully normalize driving competence [6,12]. Second-generation antipsychotics such as aripiprazole and paliperidone have been linked to higher driving-related cognitive scores in certain observational studies [6]. In mood disorders, adherence to antidepressants or mood stabilizers has been associated with differences in driving-related outcomes, whereas discontinuation or use of sedating agents may increase crash risk [11,30,31].

Occupational therapists and specialized driving assessors may complement cognitive evaluations with on-road assessments, capturing behavioral tendencies such as impulsivity, fatigue, and risk perception [3,32]. Such multidimensional assessment approaches align with models of adaptive self-regulation, whereby individuals adjust driving exposure or modify driving habits in response to perceived functional limitations [32]. Similar compensatory patterns have been documented in neurocognitive populations, including dementia-related conditions, illustrating how behavioral adaptation may support continued participation despite vulnerability [33]. Beyond assessment, emerging evidence suggests that occupational therapy interventions can enhance cognitive functioning in individuals with schizophrenia [34]. In parallel, studies in populations with cognitive impairment indicate that occupational therapy-based driving interventions may improve on-road performance and prolong safe driving participation [35]. Recent pilot findings further suggest that stable individuals with schizophrenia may demonstrate short-term improvements in simulator-based driving performance following targeted training; however, longitudinal effectiveness and real-world generalizability remain to be established [36]. Overgeneralization of impairment risks reinforcing stigma and restricting autonomy, whereas under-recognition of impairment may compromise safety. Balanced and individualized interpretation of evidence is therefore ethically imperative.

### 4.5. Limitations

Several considerations temper the interpretation of these findings. As a scoping review, the present study was designed to map and organize the available evidence rather than to evaluate intervention effectiveness, establish causal relationships, or generate pooled effect estimates. The breadth of methodological approaches, outcome definitions, and study designs limited the feasibility of quantitative synthesis and supports the exploratory and conceptual nature of this review.

Many of the included studies were cross-sectional or conducted under controlled experimental conditions with relatively small samples. Simulator-based and neuroimaging findings, although informative, were derived from structured laboratory paradigms that do not fully replicate the complexity and unpredictability of real-world driving. Factors such as simulator adaptation effects, potential simulator-related constraints, multiple statistical testing, and the absence of longitudinal follow-up constrain conclusions regarding predictive validity for real-world crash outcomes. It should also be acknowledged that several studies relied on expert-based classifications of driving fitness. While clinically meaningful, such determinations may not reflect standardized decision criteria, documented inter-rater reliability, or alignment with formal regulatory standards. These outcomes are therefore best understood as contextual clinical judgments rather than objective or legally validated indicators of driving competence.

Associations observed between antipsychotic treatment and driving-related cognitive performance must likewise be interpreted cautiously. Observational designs are inherently vulnerable to indication bias and residual confounding, as prescribing patterns often reflect illness severity, treatment response, comorbidities, polypharmacy, and baseline functional status. Under such conditions, medication-specific effects cannot be clearly disentangled from underlying clinical characteristics. Finally, the restriction to English-language peer-reviewed publications may have introduced language bias and limited the inclusion of potentially relevant findings reported in other languages.

## 5. Conclusions

This scoping review mapped the available evidence on the cognitive, pharmacological, and functional determinants of driving ability in individuals with schizophrenia. The findings suggest that driving-related functioning in this population is characterized by substantial heterogeneity and appears to be shaped by interacting cognitive, clinical, and contextual factors rather than being uniformly impaired. Deficits in attention, executive function, visual perception, and psychomotor speed have been associated with differences in simulated and clinical driving measures, whereas clinical stability, preserved insight, and self-regulatory behaviors have been linked in some studies to more favorable driving-related outcomes. These domains were identified across experimental, clinical, and population-based study designs, reflecting distinct but related levels of driving-related functioning. Pharmacological treatment represents a potentially relevant contextual factor within this framework. Antipsychotic adherence and certain medication regimens have been associated with differences in cognitive and driving-related measures; however, these findings derive primarily from observational designs and should not be interpreted as evidence of medication-specific causality or as a basis for treatment selection decisions.

The emerging driving profile described in this review is conceptualized as a multidimensional and conditional framework that organizes interacting determinants across cognitive capacity, treatment characteristics, and functional adaptation. Rather than defining a single driving phenotype, this framework accommodates inter-individual variability and contextual influences. Importantly, the current literature remains methodologically heterogeneous, with variability in outcome definitions, study designs, and levels of ecological validity. Recognizing these interacting domains may assist clinicians in structuring individualized evaluations within appropriate regulatory contexts, while acknowledging that formal fitness-to-drive decisions require comprehensive and case-specific assessment. Further longitudinal, multimodal, and ecologically valid studies are required to clarify predictive validity and to refine this conceptual framework in real-world settings.

## Figures and Tables

**Table 1:** Characteristics of studies included for analysis (*N* = 11).

Author (Year)	Design/Methods	Sample	Focus	Driving-Related Measures	Key Findings
Steinert et al. (2015) [9]	Cross-sectional interviews (questionnaire and cognitive test)	150 patients with schizophrenia/schizoaffective disorder	Active driving and predictors	License possession, driving activity, accident history; Trail Making Test A, GAF.	Participation: 64% licensed; 32% active drivers. Cognitive/Functional correlates: higher TMT-A, higher GAF, fewer hospitalizations, no substance use.
Brunnauer et al. (2016) [8]	Cross-sectional survey (structured questionnaire)	1.497 psychiatric inpatients (incl. schizophrenia) and 313 neurological controls	Driving participation and licensing	Self-reported license status, active driving, and car use.	Participation: lower licensing (67% vs. 89% controls); lowest rates in schizophrenia subgroup. Demographic/Clinical correlates of driving cessation: female sex, older age, pension status, schizophrenia diagnosis.
Segmiller et al. (2017) [29]	Comparative study (Wiener Test system psychomotor battery)	13 first-episode (FES) schizophrenia inpatients, 13 recurrent-episode (RES) schizophrenia inpatients, 20 HCs	Psychomotor driving skills in unmedicated schizophrenia	WTS: Visual Perception (TAVT-MB), Reactivity (DT), Concentration (COG), Vigilance (VIGIL); PANSS.	Cognitive/Psychomotor impairment: 58% impaired (WTS criteria). Additional deficit: visual perception (first-episode). No association: medication status, PANSS severity.
Fuermaier et al. (2019) [16]	Experimental study with driving simulator and neuropsychological battery.	31 schizophrenia patients; 31 healthy controls.	Cognitive correlates of simulated driving.	Driving simulator: collisions, reaction time, lane control, merging; neuropsychological tests-attention, inhibition, planning, processing speed.	Behavioral (simulator): slower speed; merging difficulty; no increased collisions. Cognitive correlates: reduced attention; impaired inhibition/planning.
Hellinger et al. (2019) [28]	Cross-sectional study (neuropsychological and clinical assessment)	60 schizophrenia outpatients (31 with license, 29 without).	Clinical, cognitive, socio-demographic predictors of license possession	License status; WCST, PANSS, MMSE; hospitalizations, employment.	Participation: license holders vs. non-drivers. Cognitive/Clinical correlates: higher MMSE, better WCST, fewer hospitalizations, lower general/negative symptoms.
Noh et al. (2020) [6]	Cross-sectional neurocognitive assessment	102 adults with schizophrenia on antipsychotic monotherapy (haloperidol, risperidone, olanzapine, aripiprazole, paliperidone)	Effect of antipsychotic type on driving-related cognition	CPAD battery: visual perception, attention, working memory, inhibitory control; TMT-B, digit span.	Fitness classification: 63% fit. Medication comparison (observational): higher competence with aripiprazole (75%), paliperidone (82%) vs. haloperidol (33%), risperidone (57%). Cognitive differences across regimens.
Biedermann et al. (2022) [7]	Naturalistic, cross-sectional study	50 clinically stable outpatients with chronic schizophrenia	Driving fitness and clinical correlates.	PANSS (Wallwork/Fortgang), MSAS (EPS), Vienna Test System, expert rating.	Clinical fitness: 44% competent; 20% partial; 36% incompetent. Negative correlates: EPS, older age, higher antipsychotic dosage. Residual symptom: disorganization.
Okada et al. (2023) [14]	Experimental neuroimaging study	20 people with schizophrenia (PWS) + 20 healthy controls (HCs)	Prefrontal activation patterns and potential driving difficulties	Driving simulator (braking, curves); fNIRS of DLPFC activation.	Behavioral (simulator): no performance differences. Neurophysiological: reduced left DLPFC activation; slower reaction times.
Okada et al. (2024) [18]	Cross-sectional multimodal study	42 schizophrenia patients (licensed drivers).	Cognitive, neural, and behavioral correlates of driving.	TMT-A/B, WMS-R, Zoo Map Test, PANSS; fNIRS; simulator: brake reaction, steering, SDLP, hazard prediction.	Behavioral (simulator): braking, hazard perception deficits. Cognitive predictors: processing speed, memory, planning. Exploratory classification: ~79% accuracy (within sample).
Staples et al. (2024) [5]	Population-based retrospective cohort study	808.432 licensed drivers; 2.551 with schizophrenia	Crash responsibility and medication adherence	Crash data (police), validated responsibility tool; Medication Possession Ratio (MPR).	Safety outcome: higher crash responsibility (aOR 1.67). Medication adherence (MPR ≥ 0.8): not protective.
Staples et al. (2025) [4]	Population-based case-crossover study	1.130 crashes involving drivers with schizophrenia	Antipsychotic adherence and crash risk.	Police crash data; MPR 30 days pre-crash vs. control interval.	Safety outcome: perfect adherence (MPR ≈ 1.0) associated with reduced crash risk (aOR 0.50); effect consistent across sex, age, and substance use.

**Table 2:** Key domains, determinants and driving-related impact in individuals with schizophrenia.

Domain	Key Determinants	Driving-Related Impact	Representative Studies
Cognitive	Attention, executive function, processing speed, working memory, visual perception	Structured driving performance (lane control, hazard perception, reaction time, braking efficiency, merging behavior)	Okada et al. [14]; Fuermaier et al. [16]; Okada et al. [18]; Hellinger et al. [28]; Segmiller et al. [29]
Psychomotor	Reaction speed, vigilance, motor coordination	Psychomotor driving performance (fitness indices, response to critical events)	Biedermann et al. [7]; Fuermaier et al. [16]; Segmiller et al. [29]
Clinical	Symptom stability, illness stage, hospitalization history	Driving participation and fitness classification (license possession, context-dependent fitness-to-drive judgments)	Biedermann et al. [7]; Brunnauer et al. [8]; Steinert et al. [9]; Hellinger et al. [28]; Segmiller et al. [29]
Pharmacological	Antipsychotic type, dosage, extrapyramidal symptoms	Cognitive and psychomotor performance indicators; clinical competence ratings	Noh et al. [6]; Biedermann et al. [7]
Treatment adherence	Medication Possession Ratio (MPR), consistency of antipsychotic treatment	Safety outcomes (crash responsibility, real-world crash risk)	Staples et al. [4]; Staples et al. [5]
Functional and behavioral	Insight, self-regulation, compensatory strategies	Adaptive driving behavior and risk mitigation	Steinert et al. [9]; Fuermaier et al. [16]; Okada et al. [18]
Neurophysiological	Dorsolateral prefrontal cortex activation (fNIRS)	Neurocognitive correlates of structured driving performance (reaction time, hazard perception under load)	Okada et al. [14]; Okada et al. [18]

## Data Availability

Not applicable. All data analyzed in this study are from previously published sources.

## Supplementary Materials

The following supporting information can be downloaded at: https://www.mdpi.com/article/10.3390/neurolint18030046/s1, Supplementary File S1: PRISMA-ScR Checklist.

## Abbreviations

The following abbreviations are used in this manuscript:

aOR	Adjusted Odds Ratio
COG	Vienna Concentration Test
CPAD	Computerized Psychomotor and Attention Diagnostic Battery
DLPFC	Dorsolateral Prefrontal Cortex
DT	Vienna Determination Test
EEG	Electroencephalography
EPS	Extrapyramidal Symptoms
fNIRS	Functional Near-Infrared Spectroscopy
GAF	Global Assessment of Functioning
HCs	Healthy Controls
ICF	International Classification of Functioning Mini-Mental State Examination
MMSE	
MPR	Medication Possession Ratio
MSAS	Modified Simpson–Angus Scale
PANSS	Positive and Negative Syndrome Scale
PCC	Population–Concept–Context
PRISMA-ScR	Preferred Reporting Items for Systematic Reviews and Meta-Analyses Extension for Scoping Reviews
SDLP	Standard Deviation of Lateral Position
TAVT-MB	Vienna Test System for Traffic and Motor Skills
TMT-A/TMT-B	Trail Making Test, Part A/Part B
VIGIL	Vienna Vigilance Test
WCST	Wisconsin Card Sorting Test
WMS-R	Wechsler Memory Scale-Revised
WTS	Vienna Test System
