# Supplementary material for: The Driving Profile of Individuals with Schizophrenia: Cognitive Characteristics, Pharmacological Treatment and Driving Competence—A Scoping Review
Source: Neurol Int. 2026 Feb 28;18(3):46. doi: 10.3390/neurolint18030046 (PMC13029326; doi:10.3390/neurolint18030046)
Supplement: Supplementary file 1 [file neurolint-18-00046-s001.zip › neurolint-4111870-supplementary.pdf]

Preferred Reporting Items for Systematic reviews and Meta-Analyses extension for Scoping Reviews (PRISMA-ScR) Checklist

SECTION	ITEM	PRISMA-ScR CHECKLIST ITEM	REPORTED ON PAGE #
**TITLE**			
Title	1	The Driving Profile of Individuals with Schizophrenia: Cognitive Characteristics, Pharmacological Treatment and Driving Competence - A Scoping Review	1
**ABSTRACT**			
Structured summary	2	**Abstract** **Background/Objectives**: Limited evidence exists regarding driving in schizophrenia. The aim of this paper was to map and describe the existing evidence defining the driving profile in schizophrenia by delineating the cognitive, pharmacological and functional factors underlying driving competence in this population and by synthesizing findings from experimental, neurocognitive, and population-based studies. **Methods:** Following the PRISMA-ScR (Preferred Reporting Items for Systematic Reviews and Meta-Analyses Extension for Scoping Reviews) guidelines, a structured search of PubMed and Scopus was conducted. The inclusion criteria were: i) original studies involving individuals with schizophrenia, ii) evaluating at least one of the three driving-related dimensions-cognitive, pharmacological, functional determinants-, iii) published between 2015 and 2025. Extracted data included sample cognitive, pharmacological and functional characteristics. A narrative and thematic synthesis was then performed. **Results**: Eleven eligible studies met the inclusion criteria. Findings were grouped into three categories: (1) cognitive domains: attention, executive function, reaction, visuospatial ability, (2) pharmacological influences: drug comparison, dosage impact, side effects, treatment stability, treatment comparisons, (3) functional capacity and self-regulation: data on licenses, active drivers, avoidance behaviors, self-regulation, abstinence from driving, and social adaptation. **Conclusions:** This scoping review synthesizes and integrates current evidence on the multi-dimensional driving profile of individuals with schizophrenia, highlighting the interplay between cognitive inefficiencies, pharmacological modulation and compensatory self-regulation. Understanding the interaction between these domains provides a foundation for individualized fitness-to-drive assessments, targeted rehabilitation strategies, and the development of evidence-based mobility policies in this population.**Keywords:** schizophrenia; driving; profiling; medication adherence; cognitive performance; functional capacity	1
**INTRODUCTION**			
Rationale	3	The present scoping review aims to delineate and map the cognitive, pharmacological, and functional profile underlying driving competence in individuals with	2

St.Michael's
Inspired Care.
Inspiring Science.

SECTION	ITEM	PRISMA-ScR CHECKLIST ITEM	REPORTED ON PAGE #
		schizophrenia, by synthesizing findings from experimental, neurocognitive, and population-based studies. This approach was chosen because the literature is heterogeneous in design and scope and a scoping review allows comprehensive mapping of the evidence and identification of knowledge gaps to inform clinical assessment and fitness-to-drive evaluation.	
Objectives	4	The scoping review aimed to address the following research questions:<br><br>Which cognitive, pharmacological, and functional domains collectively define the driving profile of individuals with schizophrenia?<br><br>How do these domains interact to influence driving competence, including both simulated and real-world outcomes?<br><br>What methodological or conceptual gaps remain in defining this profile across the existing literature?<br><br>Key elements considered include: the population (individuals with schizophrenia), concepts (cognitive, pharmacological, and functional determinants of driving competence), and context (laboratory, simulator, clinical and population-level studies).	3
**METHODS**			
Protocol and registration	5	No prior review protocol was registered for this scoping review. The review was conducted according to the PRISMA-ScR guidelines.	3
Eligibility criteria	6	Studies were included according to the Population–Concept–Context (PCC) framework:<br><br>Population: Adults diagnosed with schizophrenia, irrespective of illness duration or treatment setting.<br><br>Concept: Driving ability or driving-related competencies, including real-world indicators, simulator performance, neuropsychological tests, self-reported driving behavior, and neuroimaging measures.<br><br>Context: Research examining clinical, cognitive, pharmacological, or functional factors influencing driving, including the effects of antipsychotic medication, side effects, and adherence.<br><br>Only peer-reviewed studies published in English were eligible. All study designs were considered, provided they contained relevant data for mapping the driving profile in schizophrenia.	3
Information sources*	7	Studies were identified through systematic searches of PubMed, Scopus, and ScienceDirect, covering the period from January 2015 to September 2025. Additional sources included reference lists of included studies. No direct	3

SECTION	ITEM	PRISMA-ScR CHECKLIST ITEM	REPORTED ON PAGE #
		contact with authors was performed. The most recent search was executed in September 2025.	
Search	8	The full electronic search strategy for PubMed (adapted to each database) included: ("schizophrenia" OR "psychosis") AND ("driving competence" OR "fitness to drive") AND ("cognitive function" OR "executive function" OR "attention" OR "visual perception" OR "psychomotor performance") AND ("simulator" OR "medication adherence" OR "side effects" OR "antipsychotic treatment"). Limits: English language, publication date 2015–2025.	3
Selection of sources of evidence†	9	Titles and abstracts were screened for relevance, followed by full-text assessment of potentially eligible studies. Discrepancies were resolved through discussion among reviewers. The process is illustrated in the PRISMA-ScR flow diagram (Figure 1).	3,4
Data charting process‡	10	Data were charted using structured extraction tables designed and piloted by the review team. Data extraction was performed independently by two reviewers, and discrepancies were resolved through discussion. Extracted items included study design, sample characteristics, driving-related outcomes, and cognitive, clinical, pharmacological, and functional indicators.	3, 4, 5
Data items	11	Variables extracted included: study design, sample size, demographic and clinical characteristics, driving-related outcomes, cognitive measures, pharmacological treatment and functional indicators.	5
Critical appraisal of individual sources of evidence§	12	No formal critical appraisal was conducted, as the aim of this scoping review was to map available evidence rather than assess study quality.	Click here to enter text.
Synthesis of results	13	Data were collated, summarized, and synthesized thematically across four domains: (a) clinical and behavioral predictors of driving competence, (b) cognitive and neuropsychological correlates, (c) pharmacological and treatment-related influences, and (d) functional capacity, self-regulation, and real-world driving outcomes. Results are presented narratively and in structured tables to enable comparison across studies.	3,4
**RESULTS**			
Selection of sources of evidence	14	A total of 1,389 records were identified through database searches (PubMed, Scopus, and ScienceDirect). After removing 220 duplicates, 1,169 records were screened, and 1,052 were excluded as not relevant. Of the 117 full-text reports assessed, 109 were retrieved and evaluated for eligibility, with 98 excluded for not meeting inclusion criteria. Finally, 11 studies were included in the scoping review. The selection process is illustrated in Figure 1 (PRISMA flow diagram).	4
Characteristics of sources of evidence	15	The included studies spanned 2015–2025, with study designs including cross-sectional surveys and interviews, comparative and experimental studies with neuropsychological and simulator-based assessments,	5,6

SECTION	ITEM	PRISMA-ScR CHECKLIST ITEM	REPORTED ON PAGE #
		and population-based cohort/case-crossover analyses. Sample sizes ranged from 13–808,432 participants. Data charted included study design, sample characteristics, driving outcomes, cognitive, pharmacological, and functional measures. Detailed characteristics and key findings are summarized in Table 1.	
Critical appraisal within sources of evidence	16	No formal critical appraisal of individual sources was conducted, as this scoping review aimed to map existing evidence rather than assess study quality.	Click here to enter text.
Results of individual sources of evidence	17	Each included study contributed data on cognitive, clinical, pharmacological, or functional determinants of driving performance. Key findings included impairments in visual perception, attention, executive function, and psychomotor skills in schizophrenia patients; differential effects of antipsychotic medications on cognitive competence and driving fitness; reduced rates of license possession and active driving; and elevated crash responsibility mitigated partially by treatment adherence and symptom stability.	4-8
Synthesis of results	18	Data were synthesized thematically across four main domains: (a) cognitive, psychomotor, and functional performance; (b) clinical and pharmacological correlates of driving participation; (c) population-level crash risk and real-world safety; and (d) combined influence of cognitive, clinical, and functional factors on driving competence. Narrative synthesis, structured tables (Table 1), and flow diagrams (Figure 1) summarize findings in relation to the review questions and objectives.	4-8
**DISCUSSION**			
Summary of evidence	19	This scoping review synthesized evidence from 11 studies published between 2015 and 2025 on the cognitive, clinical, pharmacological, and functional determinants of driving performance in individuals with schizophrenia. Across diverse methodologies-including surveys, neurocognitive assessments, simulator-based experiments, and population-level analyses-patients consistently exhibited deficits in attention, executive function, processing speed, visual perception, and psychomotor skills. First-episode and recurrent schizophrenia patients showed impaired reaction time, concentration, lane control, and merging ability, whereas clinically stable outpatients, particularly those on second-generation antipsychotics, demonstrated higher driving competence. Cognitive, pharmacological, and functional factors interacted to shape driving performance, and adherence to treatment, symptom stabilization, and compensatory self-regulatory behaviors mitigated risk. Findings were consistent with previous literature on driving competence in psychiatric populations and provide a framework for clinical assessment and intervention.	9
Limitations	20	Limitations of this scoping review include heterogeneity in study designs, small sample sizes in some experimental studies, variability in driving assessment methods, and limited generalizability due to language restrictions	9,10

SECTION	ITEM	PRISMA-ScR CHECKLIST ITEM	REPORTED ON PAGE #
		(English only). The lack of formal critical appraisal of included studies may limit the ability to draw strong conclusions regarding evidence quality. Furthermore, some population-level studies relied on administrative data, which may not capture all relevant functional or cognitive variables influencing driving competence.	
Conclusions	21	The evidence indicates that individuals with schizophrenia exhibit a distinct driving profile influenced by cognitive, clinical, pharmacological, and functional factors. Clinically stable patients on second-generation antipsychotics may maintain safe driving competence, whereas deficits in attention, executive function, and psychomotor performance are associated with reduced driving ability. Comprehensive assessments integrating cognitive testing, functional capacity, and treatment adherence are recommended for evaluating fitness-to-drive. Occupational therapy interventions and specialized driving assessments may support safer driving practices. Future research should address methodological heterogeneity, explore long-term driving outcomes, and develop targeted interventions to optimize driving safety in this population.	10, 11
**FUNDING**			
Funding	22	This scoping review did not receive any external funding.	Click here to enter text.

JBI = Joanna Briggs Institute; PRISMA-ScR = Preferred Reporting Items for Systematic reviews and Meta-Analyses extension for Scoping Reviews.
* Where *sources of evidence* (see second footnote) are compiled from, such as bibliographic databases, social media platforms, and Web sites.
† A more inclusive/heterogeneous term used to account for the different types of evidence or data sources (e.g., quantitative and/or qualitative research, expert opinion, and policy documents) that may be eligible in a scoping review as opposed to only studies. This is not to be confused with *information sources* (see first footnote).
‡ The frameworks by Arksey and O'Malley (6) and Levac and colleagues (7) and the JBI guidance (4, 5) refer to the process of data extraction in a scoping review as data charting.
§The process of systematically examining research evidence to assess its validity, results, and relevance before using it to inform a decision. This term is used for items 12 and 19 instead of "risk of bias" (which is more applicable to systematic reviews of interventions) to include and acknowledge the various sources of evidence that may be used in a scoping review (e.g., quantitative and/or qualitative research, expert opinion, and policy document).

*From:*Tricco AC, Lillie E, Zarin W, O'Brien KK, Colquhoun H, Levac D, et al. PRISMA Extension for Scoping Reviews (PRISMAScR): Checklist and Explanation. Ann Intern Med. 2018;169:467–473.doi: 10.7326/M18-0850.
